# Mapping estimates of vascular permeability with a clinical indocyanine green fluorescence imaging system in experimental pancreatic adenocarcinoma tumors

**DOI:** 10.1117/1.JBO.28.7.076001

**Published:** 2023-07-13

**Authors:** Matthew S. Reed, Marien Ochoa, Kenneth M. Tichauer, Ashley Weichmann, Marvin M. Doyley, Brian W. Pogue

**Affiliations:** a University of Wisconsin-Madison, Department of Medical Physics, Madison, Wisconsin, United States; b Illinois Institute of Technology, Department of Biomedical Engineering, Chicago, Illinois, United States; c University of Rochester, Department of Electrical and Computer Engineering, Rochester, New York, United States

**Keywords:** indocyanine green, surgery, tumor, imaging systems, angiogenesis, fluorescence

## Abstract

**Significance:**

Pancreatic cancer tumors are known to be avascular, but their neovascular capillaries are still chaotic leaky vessels. Capillary permeability could have significant value for therapy assessment, and its quantification might be possible with macroscopic imaging of indocyanine green (ICG) kinetics in tissue.

**Aim:**

The capacity of using standard fluorescence surgical systems for ICG kinetic imaging as a probe for capillary leakage was evaluated using a clinical surgical fluorescence imaging system, as interpreted through vascular permeability modeling.

**Approach:**

Xenograft pancreatic adenocarcinoma models were imaged in mice during bolus injection of ICG to capture the kinetics of uptake. Image analysis included ratiometric data, normalization, and match to theoretical modeling. Kinetic data were converted into the extraction fraction of the capillary leakage.

**Results:**

Pancreatic tumors were usually less fluorescent than the surrounding healthy tissues, but still the rate of tumor perfusion could be assessed to quantify capillary extraction. Model simulations showed that flow kinetics stabilized after about 1 min beyond the initial bolus injection and that the relative extraction fraction model estimates matched the experimental data of normalized uptake within the tissue. The kinetics in the time period of 1 to 2 min post-injection provided optimal differential data between AsPC1 and BxPC3 tumors, although high individual variation exists between tumors.

**Conclusions:**

ICG kinetic imaging during the initial leakage phase was diagnostic for quantitative vascular permeability within pancreatic tumors. Methods for autogain correction and normalized model-based interpretation allowed for quantification of extraction fraction and difference identification between tumor types in early timepoints.

## 1 Introduction

Fluorescence-guided surgery with an indocyanine green (ICG) tracer has been approved for several indications around tissue perfusion, specifically for viability of tissue flaps or for bowel anastomosis leakage detection.^1^^–^^3^ It has been explored for surgical guidance in cancer tumors, although definitive adoption for oncologic use has not been widely accepted to date because it has not been apparent if this imaging offers clinical value. The temporal aspect of the ICG flow has been examined to discriminate between healthy and diseased tissue either by the long-lived presence remaining after many hours^4^^,^^5^ or by the early phase of perfusion tracking blood flow.^6^ Most oncologic studies in this field involve the former method of imaging hours or days post-injection of ICG, but dynamic kinetics imaging has also been explored and has the benefit of immediate use so as not to disrupt clinical workflows. A number of studies have examined the phenomenological flow parameters from temporal ICG signals; a recent review listed those that had quantitative analysis of *in vivo* data.^7^ An example is analysis of the ICG fluorescence temporal kinetics in human primary colorectal cancers indicating discriminatory information about the malignancy in the slope of the uptake^8^ and benign tumors having a faster decrease after the signal apex. Similar metrics have also been interrogated to assess pancreatic tissue perfusion during pancreatectomy for the purpose of fistula prevention, utilizing fluorescence-based augmented reality, and hyperspectral imaging to track a bolus ICG injection and produce a perfusion heatmap.^9^ Model and data estimations of the flow kinetics suggest that a very important metric of tumor pathophysiology, vascular permeability, can also be quantified from ICG time kinetics.^6^ In tumors, the neovascular leakage is a known characteristic of the pathology, and the magnitude of it is related to chemotherapy and immune response potential. So, in this study, an analysis of the ability to quantify vascular leakage rates from imaging ICG fluorescence kinetics was completed using a commercially available human system.

Adopting a commercial system for testing of flow kinetics brings the distinct advantage of providing a well-engineered interface with good ergonomic use and direct clinical translation. However, the distinct disadvantage of adopting such a device is that it is not designed for flow kinetic imaging and has features that can limit the accuracy of kinetic measurement if not corrected for. Most commercial systems have a low dynamic range in the images because the bit-depth is optimized for video-rate stream imaging, meaning that the number of gray scales in the image is typically limited to 256 integer values (i.e., 0 to 255).^10^ Because of this, most systems have automatic gain range features built into them to adjust the sensitivity for different levels of light. This allows for an expansion of the effective dynamic range, but it comes at the cost of a non-linear response in intensity. In this study, this non-linear responsivity was examined and solutions to mitigate it for longitudinal temporal data were analyzed. Both external reference targets and internal reference tissue^11^^,^^12^ measurements were tested.

The value of flow imaging to quantify vascular permeability changes would be significant for a range of reasons in clinical research, such as (1) for diagnostic assessment, (2) tracking response to therapies, or (3) assessing margins of tissue in resection. In these applications, the assessment should ideally be time-efficient (i.e., not disrupt clinical workflows), and ICG kinetic imaging of vascular leakage is typically achieved within minutes. However, the variability of leakage can be high, and the ability to provide objective numbers that have a diagnostic value as a biomarker may be limited by the range of values expected and the known biological variability. So, in this study, we examined both the intratumor variability and the inter-tumor variability, using two different pancreatic cancer cell lines with known differences in vascular supply including AsPC-1 tumors with a more chaotic pattern and BxPC3 tumors with a more organized and regular perfusion pattern.^13^^–^^15^ The observed capillary permeabilization rates were interpreted with an adiabatic approximation model to homogeneous tissue leakage, in which the extraction fraction of ICG dye was quantified with a simplified model interpretation.^6^ This modeling and the potential to image capillary permeabilization rate were examined.

## 2 Materials and Methods

### 2.1 Tumor Cell Line

Both AsPC1 cells and BxPC3 cells were obtained from American Type Culture Collection (ATCC) and grown in Roswell Park Memorial Institute (RPMI) 1640 Media supplemented with 10% fetal bovine serum and 1% penicillin/streptomycin. Cells were grown in a 5% ${\mathrm{CO}}_{2}$ incubator. Athymic nude mice were injected with cells via flank injection, AsPC1 at $1\times 10^{6}\text{ cells}/\text{injection}$ and BxPC3 at $2\times 10^{6}\text{ cells}/\text{injection}$, with an injection volume of 0.1 mL. Mice were selected for imaging once tumors reached 4- to 8-mm in diameter. Tumor depth was not allowed to reach >5  mm to avoid extensive necrosis and maximize the ability to achieve near-infrared (NIR) imaging through a representative depth of the tumor.

### 2.2 Fluorescence Imaging and System Verification

**Fig. 1:**
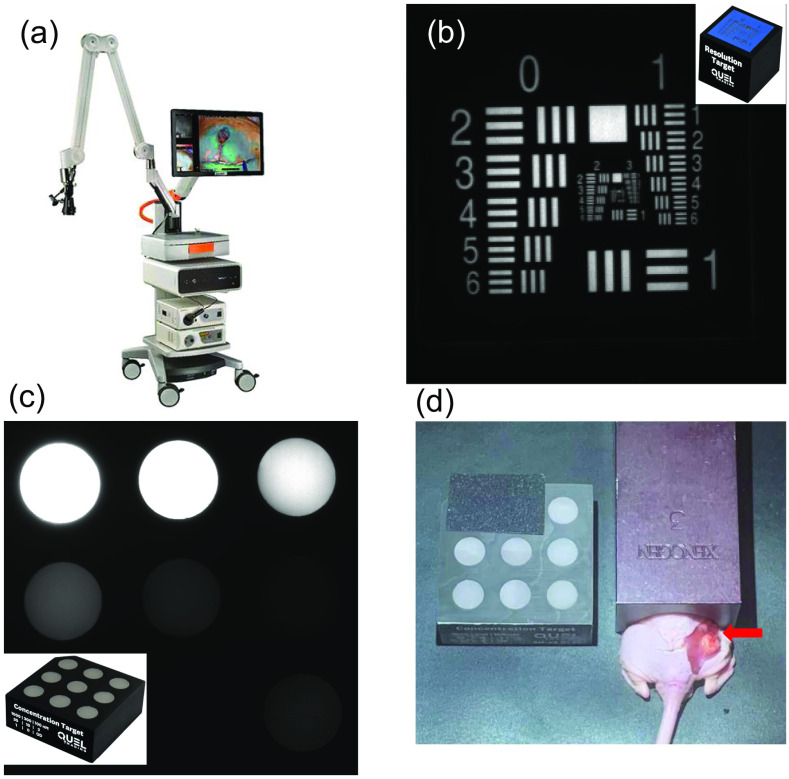
(a) EleVision NIR imaging system used (Medtronic Inc.), (b) NIR fluorescent resolution target image, with inset thumbnail image of target (QUEL Imaging LLC), (c) NIR concentration target image, with inset thumbnail image of target (QUEL Imaging LLC), and (d) mouse with exposed tumor on muscle (arrow), with abdomen covered to eliminate the dominant fluorescence from liver and kidneys from the imaging field of view.

Imaging was completed with a commercial human fluorescence surgical imaging system called the EleVision IR Platform with the Visionsense VS3 Iridium open imaging camera system (Medtronic, Minneapolis, Minnesota, United States), equipped with 785-nm laser excitation [Fig. 1(a)]. The NIR-Hi Contrast setting of the EleVision system was used in all studies; it utilizes contrast stretching to provide a better image when the signal to background ratio is low. The system has built-in automatic electronic gain variation that is always present, which adjusts the output intensity to maximize the dynamic range of imaging with the camera. This made it challenging to extract quantitative data from the system, as discussed in the next paragraph. Camera focusing was done manually using a knob located on the imaging head, and the focus accuracy was verified using a fluorescent resolution target (QUEL Imaging LLC, White River Jct, Vermont, United States), which contained a United States Air Force (USAF) resolution test chart, with background material embedded with an ICG-equivalent dye.^16^ The fluorescent lines of varying sizes could be focused down to 1.26  lp/mm, and this was used periodically through the study on different days to ensure that similar focus and spatial resolution was achieved across experiments [Fig. 1(b)].

To correct for intensity variation in the image that came from the camera system automatic gain adjustment setting, a fluorescent concentration target (QUEL Imaging LLC, White River Jct, Vermont, United States) was placed in the field of view for every trial. This target contained solid 3D-printed circular wells, each containing a different concentration of ICG-equivalent dye. Prior to imaging, one mouse was used as a test to ensure that the ICG injection dose and volume were suitable for imaging with the EleVision, as well as for selecting a well of suitable intensity on the QUEL concentration target. The upper torso of the mouse above the hind legs was covered to avoid saturation of the camera from brighter organs. In addition, only those calibration wells from the QUEL target that matched the intensity of the mouse were exposed for imaging in the field of view [Fig. 1(c)].

### 2.3 Animal Studies

Animal studies were conducted in accordance with protocols approved by the University of Wisconsin School of Medicine and Public Health Institutional Animal Care and Use Committee (Protocol M006554-A03). All efforts were made to minimize animal suffering. All surgical procedures and ICG injections were performed under isoflurane anesthesia.

Each BxPC3 mouse was imaged both with the skin entirely intact and with an incision made to expose the tumor and the muscle tissue beneath. AsPC1 mice were imaged only with the tumor and underlying muscle exposed. ICG (cat # 099555 Matrix Scientific, Columbia, South Carolina, United States) was obtained, dissolved in PBS, and diluted to a concentration of 0.35  mg/mL. A 0.1-mL injection of ICG was administered at 1  mg/kg via a tail vein injection, utilizing an injection cannula to minimize movement during the injection process. The ICG injection was followed by a 0.05-mL saline flush as this is the approximate volume of the cannula.

The laser of the EleVision system is preset to automatically turn off after 10 min, so 10 min of usable data were acquired in each trial, though ICG did not completely clear from muscle tissue in this time window. Repeated imaging sessions were completed, but most of the usable permeability data was contained within the first 1 to 8 min of imaging after ICG injection. The first minute of imaging was typically highly variable depending upon the mouse and the process of injection. After about 1 min, most mice showed a monotonic increase in overall intensity throughout most tissues at least for another minute or two, which often was followed by a region of saturation or slow clearance. The mice upper abdomen was covered, as seen in Fig. 1(d), due to excessive fluorescence coming from the kidneys and liver during the imaging sessions, which would obscure the lower-level fluorescence from the tumor region. The ICG injection occurred at 5 to 10 s into the imaging studies.

### 2.4 Image Post-Processing

**Fig. 2:**
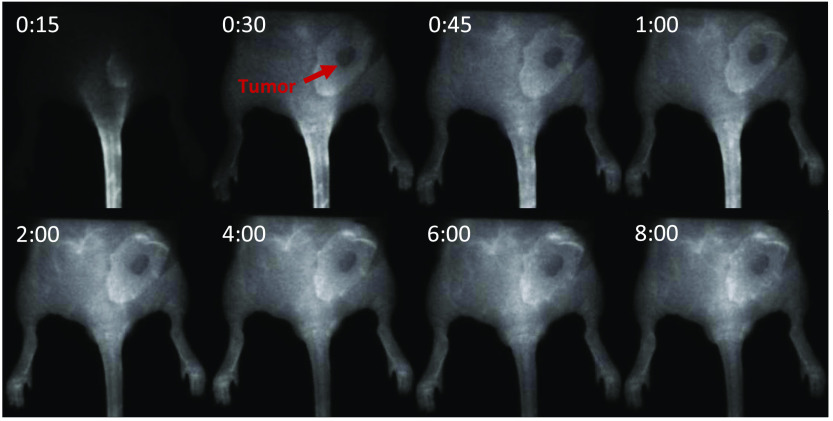
Static image time sequence of a mouse with an AsPC1 tumor. The tumor appears darker than normal tissue, but still the intensity increases over time. Each mouse was imaged for 10 min. Each image timepoint is denoted at the top in minutes and seconds.

**Fig. 3:**
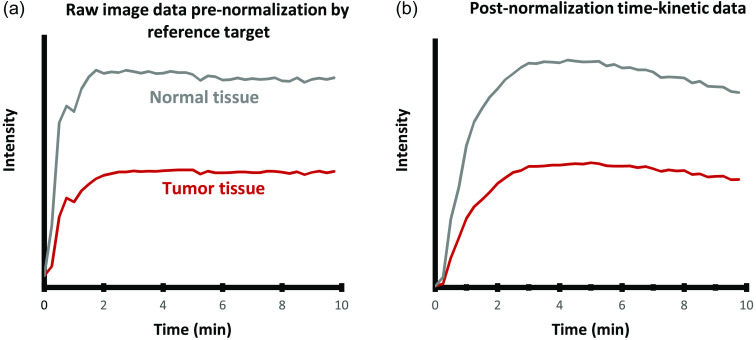
Demonstration of the normalization of ICG kinetics image data, using a fixed external fluorescent target as a reference point to correct for system gain changes. The tumor and normal tissue intensity of a single mouse with AsPC1 tumor (shown in Fig. 2) was extracted from the images over time. (a) Raw intensity data from the images, which are affected by the system’s auto-gain functionality, and (b) normalized raw data, producing accurate ICG kinetic curves.

NIR monochromatic images were taken at 15-s time intervals and uploaded into Fiji (ImageJ) as an image stack. Representative images are shown in Fig. 2 at different timepoints. Note that the tumor was commonly one of the darker parts of the mouse, likely owing to the avascular nature of pancreatic cancer with high stromal density. Region of interests (ROIs) of the 100-nM fluorophore concentration well, the mouse tumor, and the normal tissue were manually segmented in each image, and the average intensity of each ROI was taken at each timepoint for analysis. The transient ICG intensity data were acquired for each tissue, as shown in Fig. 3.

Initial imaging with normal mice showed that the intensity levels were commensurate with the 100-nM-concentration well of the calibration target, so this was deemed suitable for use as a reference target for normalizing signals. Throughout imaging, it was imperative that bright artifacts were covered up so as not to move outside the sensitivity window of the device. In this aspect, the liver and kidneys were always nearly 10× brighter as the blood pools for ICG signal in them, and they brightened almost immediately following injection and even beyond 1 h after injection and in the bladder. Thus, the mouse cover shown in Fig. 1(d) was used to cover the parts of the body over these organs. In addition, ICG particulates on gloves created artifacts when the mouse was handled, so all of these artifacts needed to be covered with a black or light-opaque material.

**Fig. 4:**
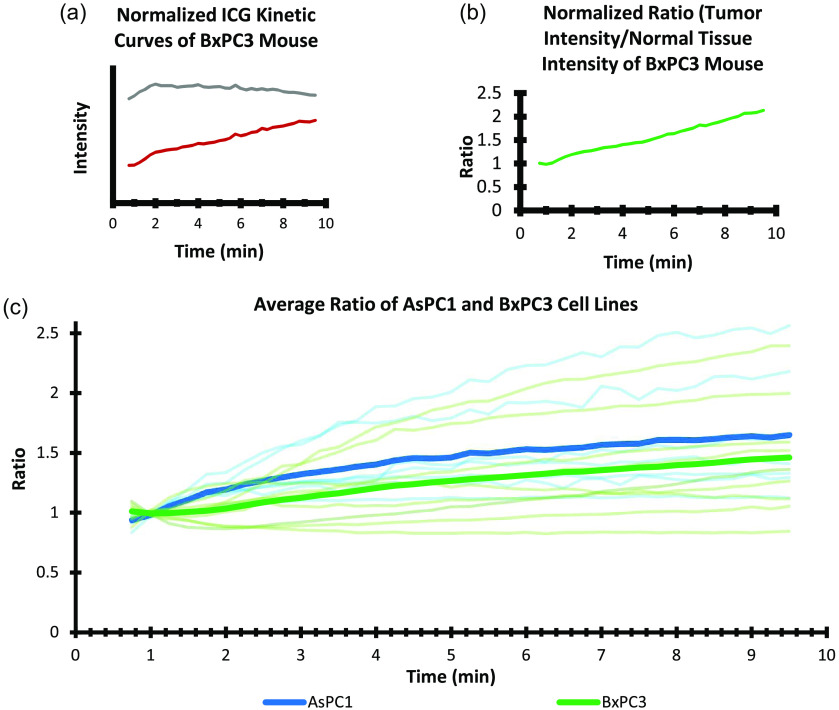
(a) Example ICG kinetic curve from BxPC3 tumor mouse. Only timepoints from 45 s to 9:30 min showed the relevant capillary leakage kinetics. These were not corrected for autogain as generation of the ratio of intensities of tumor/normal tissue was used for all mice, as shown in panel (b). (c) All data from each mouse, with normalization to the first three data timepoints in each curve. AsPC1 mice are shown in light blue, and BxPC3 are in light green, with the average ratio of intensities for AsPC1 and BxPC3 mice shown in dark blue and dark green, respectively.

Normalized intensity data from the mice could be obtained [see Fig. 3(b)] and provided absolute kinetics of the ICG from the images. However, it is well known that this normalization process does not account for animal-to-animal variations in ICG uptake and biodistribution, so interpretation of kinetics typically requires some kind of normalization to the blood plasma or other reference tissues.^11^^,^^12^ So here, a reference tissue normalization process was examined to obtain objective information about the tumors, with autologous normalization for each animal, illustrated in Fig. 4. A region of muscle tissue was chosen in post-processing for each mouse, and the average measure ICG signal intensity of tumor, $I_{T}(t)$, to normal (muscle), $I_{N}(t)$, tissue were taken as a ratio for each timepoint, i, such that the ratio, r(t) was estimated at each ROI as

$$r(t_{i})=I_{T}(t_{i})/I_{N}(t_{i}).$$ (1)

### 2.5 Theoretical Modeling of Signals

**Fig. 5:**
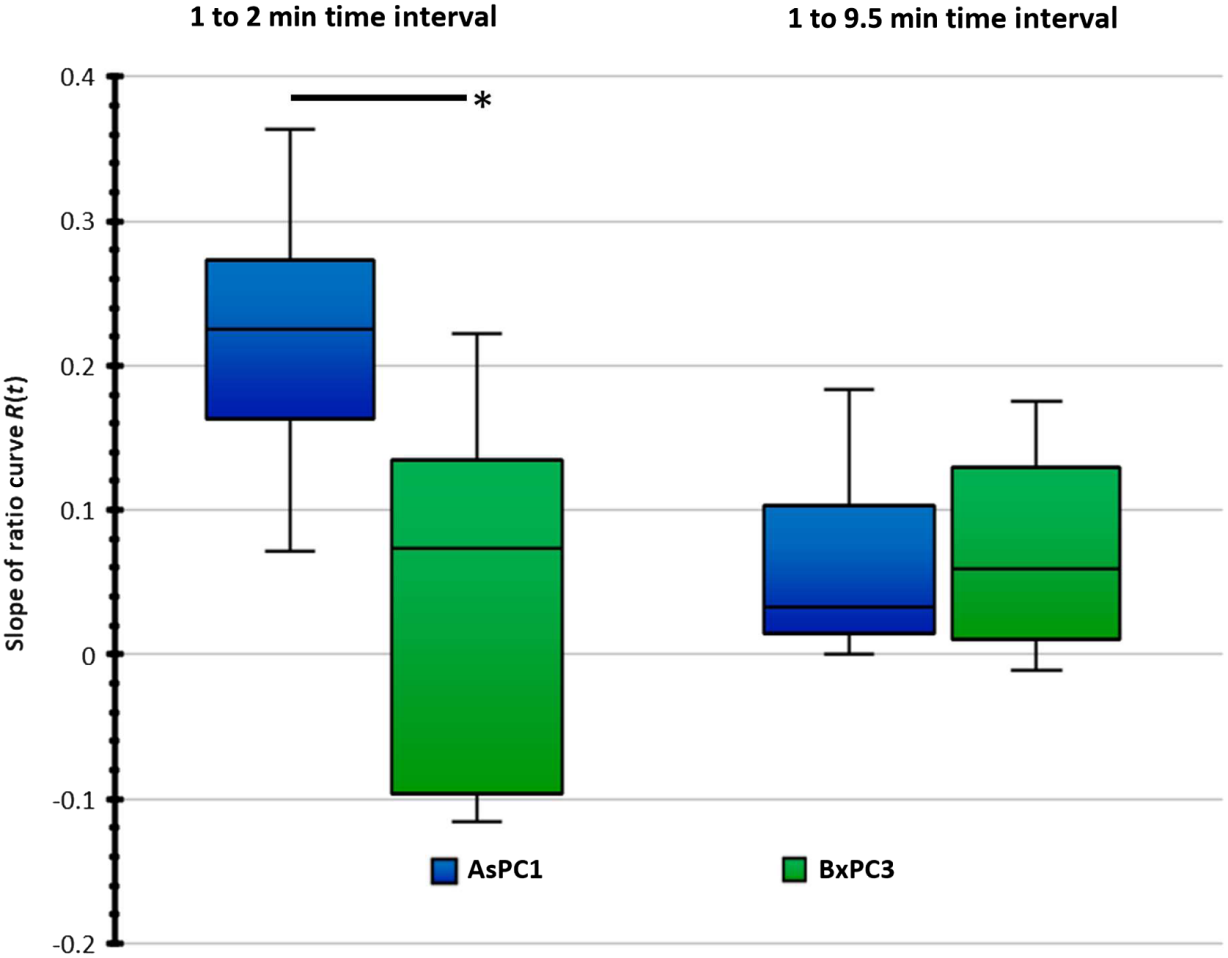
Ratio curve slopes of all individual AsPC1 and BxPC3 mice compared at two different time intervals. *Indicates statistically significant difference (P-value=0.0055).

Kinetic modeling simulations were carried out to support the belief that tumor pathophysiology characteristics, such as capillary or vascular leakage, could be driving the observed differences between individual tumors and tumor types, based upon the measured slopes of the ICG signal intensity ratio from tumor to muscle [Eq. (1)]. With the hypothesis that the initial slope of R(t) reflects the vascular permeability at that point in the tumor (i.e., tumors with leakier blood vessels will yield higher slopes, predominantly earlier in the leakage phase), the adiabatic approximation to the tissue homogeneity (AATH) model was selected as a system model, based upon previous validation of this approach.^6^^,^^17^ The AATH provides direct parameter control of vascular permeability through a unitless “extraction fraction” parameter, E, which represents the fractional amount of imaging agent that leaks out of the blood and into the tissue for a single pass of blood volume through the tissue. The AATH is a model-based formulation of the indicator dilution method presented by Meier and Zierler,^18^ which represented the concentration of dye in the tissue as a function of time, Q(t), as equivalent to the blood flow (F) multiplied by a convolution between the arterial input function (concentration of dye in the blood feeding the tissue, $C_{a}(t)$), and the so-called impulse residue function, R(t), specifically,

$$Q(t)=F\cdot C_{a}(t)*R(t),$$ (2)

where * represents the convolution operator. In the AATH, R(t) is modeled as a piecewise function as

$$R(t)=\{\begin{array}{ll} 0, & t<0\\ 1, & 0<t<t_{c}\\ Ee^{-k_{e}t}, & t_{c}<t \end{array},$$ (3)

where $t_{c}$ represents the mean capillary transit time and $k_{e}$ represents the “efflux” rate constant governing transport of the dye from the tissue to blood. A total of 400 simulations were carried out, with each simulation being built from randomly select tumor values of F between 20 and 40  mL/min/100  g, $t_{c}$ between 8 and 11 s, permeability-surface area product (PS) between 0.2 and 2.2  mL/min/100  g, and tissue-to-blood “efflux” rate constant ($k_{e}$) between 0.012 and $0.012\;\min^{-1}$; and muscle values of F between 10 and 12  mL/min/100  g, $t_{c}$ between 6 and 8 s, PS between 0.1 and 0.12  mL/min/100  g, and $k_{e}$ between 0.006 and $0.012\;\min^{-1}$. These ranges were selected based on previous values obtained from mice using ICG as the contrast agent.^6^ E was calculated as ${1-e}^{-PS/F}$ [Fig. 5(a)].^6^ A single pass of the arterial input function (i.e., no recirculation) was modeled as a gamma variate function as follows:^19^

$$\mathrm{GV}=c_{1}t^{c_{2}}e^{-c_{3}t},$$ (4)

where $c_{1}$, $c_{2}$, and $c_{3}$ are constants determined by fitting Eq. (3) to the first pass of a typical mouse ICG arterial curve.^20^ Recirculations (i.e., consecutive passes of the blood through the circulatory system) were then modeled by adding consecutive convolutions of the first pass with all previous signal as follows:

$$C_{a}(t)=\mathrm{GV}+\mathrm{GV}*\mathrm{GV}+\mathrm{GV}*\mathrm{GV}*\mathrm{GV}+\ldots ,$$ (5)

for 40 recirculations and then cutting the result to 10 min (the duration of imaging in the current study). On each iteration of the simulation, $c_{1}-c_{3}$ were selected randomly from a 10% range about values chosen from the data fit. The same $C_{a}(t)$ was used for both the tumor and muscle on every simulation. Poisson (shot) noise was then added to the resulting tumor and muscle simulated curves [$Q_{\text{tum}}(t)$ and $Q_{\text{mus}}(t)$, respectively], assuming that the highest of the tumor or muscle signal was scaled to 80% of the range of a 12-bit dynamic range detector. $Q_{\text{tum}}(t)$ and $Q_{\text{mus}}(t)$ were then normalized to each other at the 1-min timepoint, and the ratio was determined by dividing the normalized signals point-by-point: $Q_{\text{tum}}(t)/Q_{\text{mus}}(t)$. The slope of the ratio—which was hypothesized to be sensitive to the vascular permeability of the tumor (specifically, the extraction fraction, E)—was then determined by least squares fitting a first-order polynomial to the ratio data from 1 to 2 min.

## 3 Results

Imaging studies with the automatic gain variation in intensity made it necessary to use a standard reference target, so repeated imaging data could be adjusted in post processing to normalize all images in terms of intensity to the reference standard (Fig. 1). Figure 2 presents typical frames from a repeated imaging study after post-processing, allowing relative time-courses of ICG kinetic curves that are insensitive to the auto-gaining camera system to be extracted. However, the ideal reference target for normalization purposes was one that displayed an intensity just below that of the brightest portion of the mouse tissue because the camera uses the brightest item in the imaging field to determine its gain-sensitivity window. Reference targets that are too low or too high in intensity would result in a loss of visibility in the target or the imaging subject, respectively. Unnormalized ICG kinetics curves appeared to show a steady linear decrease in ICG intensity after 2 min in most mice, which is uncharacteristic of normal ICG kinetics [Fig. 3(a)]. After normalization was done to the reference target, the ICG curves appeared to follow a more expected pharmacokinetic pattern [Fig. 3(b)].

This normalization approach with an external reference target was a way to generate the true ICG kinetic curves for tissue. However, this was also found not to be needed when a reference tissue was used for normalization, as described next. Reference tissue was chosen to be exposed muscle tissue just outside the field of the tumor. Calculation of the ratio, R(t), over time is illustrated in Figs. 4(a) and 4(b).

AsPC1 and BxPC3 cell lines were selected for comparison as they were known to have different capillary network morphologies.^15^^,^^21^ The BxPC3 tumors typically display a more homogeneous vascular pattern throughout their tumors, as compared with the AsPC1 tumors, which typically are highly heterogeneous and nodular with extensive regions of necrosis. The intensity data were measured from each animal in the tumor and reference tissue areas, and the ratio data, R(t) are plotted against time, in Fig. 4(c).

**Fig. 6:**
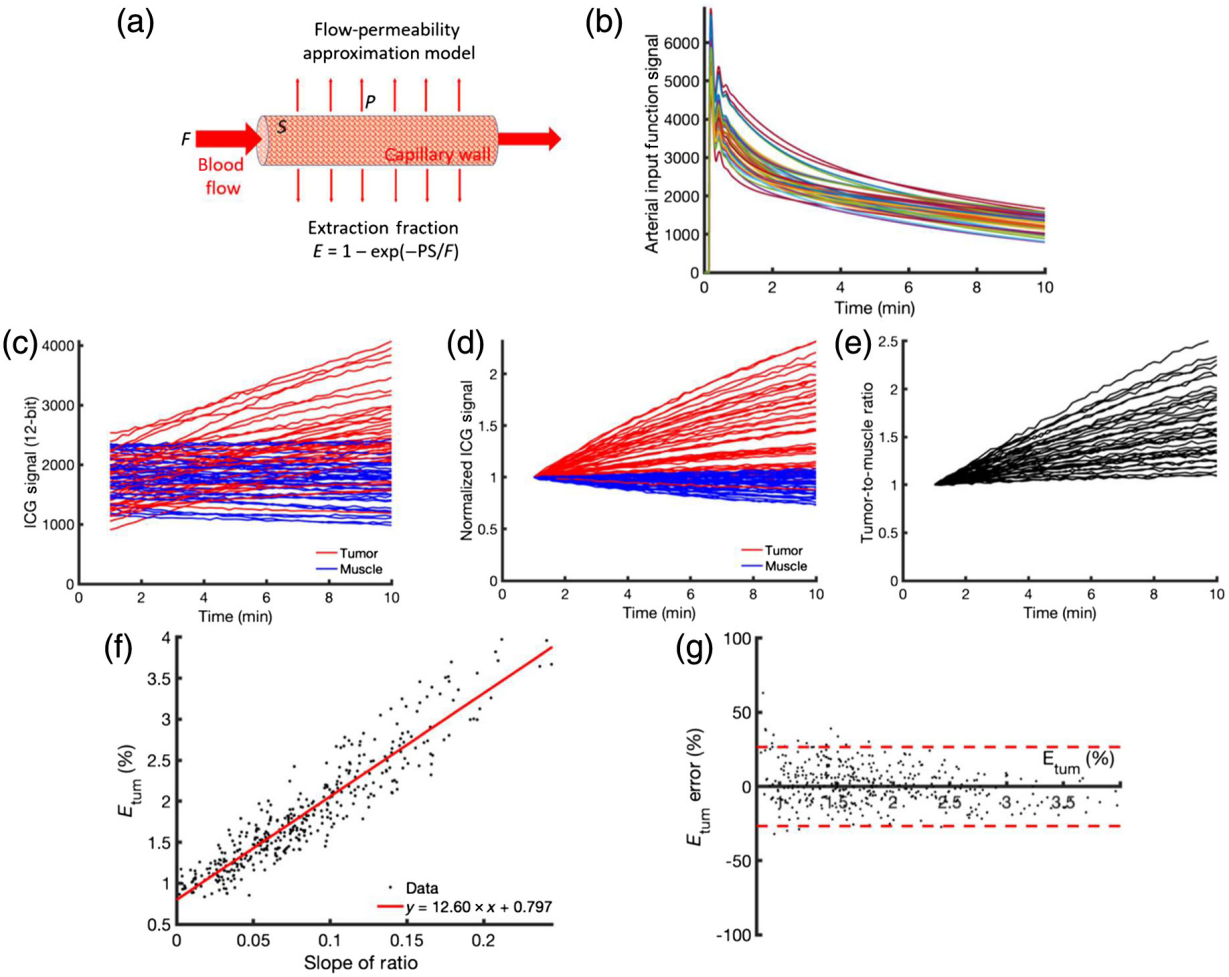
Flow-permeability model of capillary leakage is illustrated in (a) where the extraction fraction, E, is estimated based upon the permeability, P, surface area, S, and blood flow, F. Simulation of the ICG signal can be done with assumptions about the relevant values of atrial input functions (b) based upon prior studies.^6^^,^^22^ (c) Predicted range of raw ICG signals from extraction into the tissue (tumor and muscle), (d) same curves are normalized to the 1 min post-injection datapoint, and (e) ratio of these are shown normalized to the timepoint at 1 min. (f) Extraction fraction is plotted as a function of the slope of R(t) between 1 and 2 min for simulated values, showing clustering around the best fit line (red). (g) Bland-Altman plot showing 95% confidence interval in conversion of slope to $E_{\text{tum}}$ from the fitted red line in panel (e).

The temporal kinetic model is illustrated in Fig. 6, with the model basics illustrated in Fig. 5(a). Forty randomly generated arterial input functions to the model (each used in a distinct iteration of the simulation) as described in Section 2 are presented in Fig. 6(b). These were the product of introducing a 10% random variation in the 3 characteristic parameters used to simulate the curves [Eqs. (4) and (5)]. Using Eqs. (2) and (3), tumor and muscle ICG dynamic signal contrast curves were simulated [Fig. 6(c) for raw curves; Fig. 6(d) for curves normalized to a value of 1 at 1-min post simulated ICG injection]. Each simulation was developed from a unique arterial input function [e.g., Fig. 6(b)] and expected random selections of all hemodynamic and physiologic parameters as described in Section 2. Resulting ratios of tumor to muscle curves are presented in Fig. 6(e).

A strong and statistically significant correlation was observed between the simulated values of extraction fraction, E, in the simulated tumor signal ($E_{\text{tum}}$) and the slope of the ratio of tumor-to-muscle ICG signals from 1 to 2 min post-injection [p<0.01, r>0.95; Fig. 6(f)]. The correlation in Fig. 6(f) was presented such that the line-of-best-fit (y=12.6×x+0.8) could be used directly to convert experimental measurements of the slope-of-the-ratio (“x” in the equation) into estimates of the tumor extraction fraction, $E_{\text{tum}}$ (“y” in the equation) in terms of “percent extravasation of ICG for a single pass through the tissue.” Figure 6(g) provides a Bland-Altman plot depicting how precise such estimates could be based on the assumptions made in the simulations. Specifically, it is expected that 95% of the time, $E_{\text{tum}}$ estimates from experiments would be within 25% of the true value (more precisely, 95% of variance was 23.5%).

**Fig. 7:**
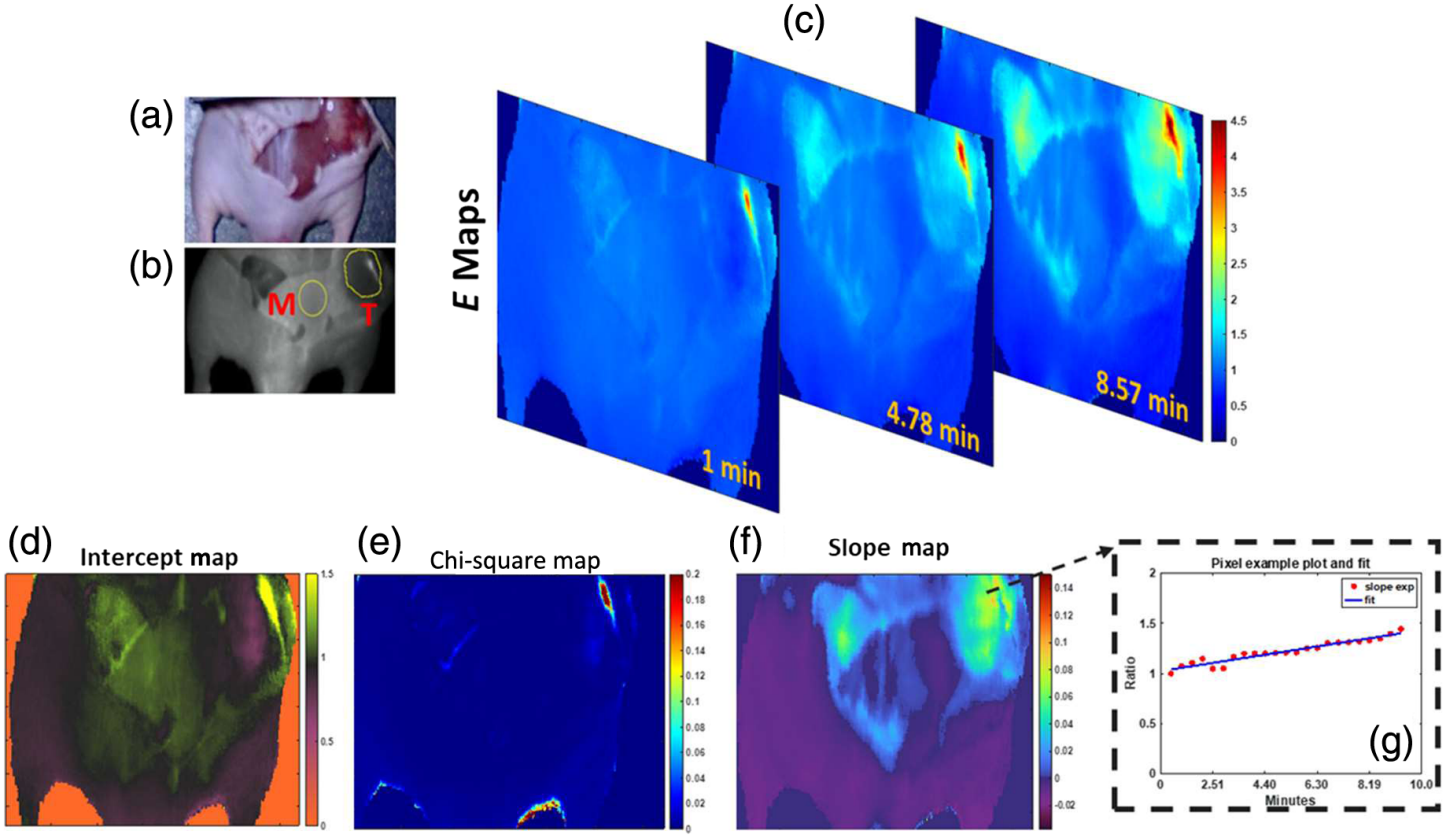
(a) Color image of exposed BxPC3 tumor. (b) Muscle “M” area used for normalization of intensity trends over time. “T” indicates the tumor region. (c) Extraction fraction maps displayed for three different timepoints after ICG injection. (f) Slope maps obtained from fitting the kinetics of ICG over time and per each pixel. (d) Intercepts resulting from the fitting process per pixel. (e) Chi-square residuals obtained from the fitting process and which are indicative of goodness of fit per each pixel of the image space. (g) Example of this fitting process for a single pixel in the tumor region is shown in plot form; both kinetics trend and estimated fit are displayed (Video 1) (Video 1, MP4, 374 KB [URL: https://doi.org/10.1117/1.JBO.28.7.076001.s1]).

The ability to create an image of the flow kinetics and the extraction fraction across the entire mouse is illustrated in Fig. 7 for an example of a mouse with a BxPC3 tumor. To estimate a map of flow kinetics, a slope and ratio model similar to the one previously reported is applied. First, each pixel’s intensity value across the mouse area is normalized to the mean intensity of a muscle region (normal tissue). An example of this region is shown in Figs. 7(a) and 7(b). Per each pixel location, intensity values per each frame acquired from 1.6 to 9 min are used, and their trend along time is subsequently fitted through a linear approximation. An example of this estimation is shown in Fig. 7(f) which gives a plot for a single pixel of the image space. The slope and intercepts calculated from the resulting fit are used to create a map of slopes and intercepts. An example of this slope map, which approximates ICG flow kinetics from 1.6 to 9 min, is shown in Fig. 7(f). Furthermore, the goodness of fit is also evaluated through the reduced Chi-square coefficient metric of the fit versus the intensity trend, which is also displayed in map form in Fig. 7(e). With the estimation of both intercept and slopes, the estimation of extraction fraction is done in correspondence to the linearization model shown in Fig. 6(e). These extraction fraction maps are then representative of the capillary leakage over time, as shown in Fig. 7(c). Of importance, this is only observed in the tumor and edges of the opened skin. Even though frames are accounted from 1 to 9.5 min, to shorten the computational time during the slope estimation process, intensity values are averaged every 100 frames or every 10 s per pixel location.

## 4 Discussion

The value of ICG NIR fluorescence imaging to quantify and track vascular permeability was examined here both experimentally and theoretically. Although the systems developed for imaging ICG fluorescence are not optimized for this functionality, it is possible to use them with the suitable approaches to referencing the signal, as examined here. The purpose of developing these methods on a commercial system was to examine if these systems were capable of estimation of extraction fraction or capillary leakage through the use dynamic imaging with appropriate normalization and model-based interpretation. The motivating purpose of this work is to assess if there is new information in this model-based flow interpretation that could have value for diagnostic or therapeutic decision making. The ability to image capillary leakage from macroscopic imaging appears to have fundamental insight, although the utility likely lies in the accuracy, specificity to tumor tissue, and sensitivity to treatment-based decisions that would change the outcome of the procedure.

The process of interpreting fluorescence kinetic data from tissue is unfortunately not as easy as it could be. The dynamic range required for imaging normal uptake and clearance can be exceptionally high,^10^ so most systems have some kind of image compression or automatic gain to adapt the camera to the average intensity of the imaging field. This process avoids saturation of the camera and provides visibility for the user. As a result, a usable approach to image processing must be developed to make useful quantitative kinetic information possible. In our first studies, we utilized a fixed reference target, which worked well to get absolute intensity correction data, as shown in Fig. 2. However, subjectively choosing a well is susceptible to human error, and the known variations in the arterial input function and physiology variations between animals means that an internal biological reference tissue is more robust for estimation of absolute parameters, such as the extraction fraction. These normalization procedures have been well developed in many previous modeling studies in MRI^19^ and translated effectively to fluorescence imaging.^6^^,^^23^^–^^25^ With this reference tissue model approach,^25^^,^^26^ it is possible to have self-normalized data from each mouse that can be applied to model-based interpretation of the data. For these reasons, this reference tissue approach was used for the remainder of the study.

The theoretical modeling completed here was done to support the understanding of the measured data and what it represents physically within the tissue microenvironment. The fact that these pancreatic adenocarcinoma tumors are highly hypovascular reduces the initial signal significantly, so the increase in signal over time is quite subtle. Still, this increase in signal is detectable and quantifiable and agrees with the modeling that suggests leakage into the tumor increases over many minutes after injection of ICG [Figs. 6(c) and 6(d)]. The correlation between the measured slope of the tumor-to-muscle ratio curves from 1 to 2 min post-injection and the model-based extraction fraction was highly significant and apparently linear [Fig. 6(f)]. This allowed the slope-of-the-ratio measurement to be converted to the predicted extraction fraction in the tumor, which simulation results suggested can be expected to be within ±23.5% of the correct extraction fraction in 95% of cases. The accuracy of using such kinetic models to verify and validate experimental methods through simulations relies heavily on the accuracy of the model used and the relevance (or physiological similarity) of model parameters and real-world parameters: namely, blood flow (F), vascular extraction fraction (E), tissue-to-blood efflux rate constant ($k_{e}$), mean transit time ($t_{c}$), and arterial input function shapes. As such, a brief discussion of how the model, parameters, and input function were selected is warranted.

In terms of the model, the AATH model^17^ has been used in several ICG kinetics studies^6^^,^^27^^,^^28^ as well as MRI studies,^19^ and importantly it provides a direct control of vascular permeability (the extraction fraction, E). Other models are likely to have been suitable—a simple one compartment “Kety” model was found to provide nearly identical results as presented with the AATH (results not shown)—each with subtle differences in what assumptions are made about the system. A detailed discussion of various models that have been used for estimating or simulating vascular permeability can be found elsewhere.^29^ The main assumptions of the AATH are that there is instantaneous mixing of the ICG once it leaves the blood and ICG transit times are all equivalent.

In terms of the model parameters and arterial input functions, an attempt was made to encompass the whole range of physiologically possible blood flows, transit times, PS, and arterial input function shapes possible for muscle and tumors, respectively (see Section 2). The relationship between slope-of-the-ratio and extraction fraction [1−exp(PS/F)] and the variance about the line-of-best-fit was found to be highly insensitive to variations in input parameters or arterial input function shapes. Instead, its accuracy was found to be dominated by noise levels. These results suggest that the slope-of-the-ratio approach can be a highly robust and simplistic way of estimating even subtle changes in vascular permeability between a tumor and a control (in this case, “muscle”) tissue. Perhaps the most important takeaway from the model-based interpretation is that the average relationship between the normalized slope of uptake of ICG in tissue versus extraction appears linear. So, if this is largely linear, then the interpretation of the slope provides a direct estimate of the extraction into the tissue with an appropriate approach to normalization.

Part of this study was to apply this imaging approach to a range of tumors to assess both the intra-tumor and inter-tumor variability present. The AsPC1 and BxPC3 pancreatic tumor cell lines were selected for comparison as they are known to display different capillary phenotypes, with BxPC3 displaying a more vascular and less stromal phenotype than AsPC1 tumors. Tumors of both cell lines appeared darker, or less fluorescent, than normal tissue immediately upon ICG injection in a majority of trials and usually remained that way for several minutes, indicating low flow and low capillary density. Lacerated mouse skin separated from muscle tissue displayed similar characteristics to tumor tissue, appearing much darker than surrounding normal tissue and usually equalizing to normal tissue over time. Because the exposition of skin tumors requires laceration and folding back of the skin, the darkness of the lacerated skin made several skin tumors difficult to visually differentiate from the skin tissue immediately surrounding it. Only skin that is pulled from the muscle tissue appears dark; skin still attached to muscle tissue appears largely normal. One AsPC1 mouse presented with a muscle tumor, and the six other AsPC1 mice presented with skin tumors. For BxPC3 mice, all tumors were muscle-bound except for one mouse with a skin tumor. The variability in normalized slope seen between tumors and between tumor lines was high, estimated to have a ±0.05 standard deviation, and the difference in early slope between the two tumor lines was 0.14 (Fig. 5).

Imaging of the tumors allows for direct full surface visualization of the tumor extraction fraction spatially across the surface, as illustrated in Fig. 7. These images provide a direct way to see the intra-tumor variation at least across the top on or two mm of the tumor tissue. Perhaps most notable from this visualization and analysis is the fact that the tumors are characterized by the trend with a low initial value [intercept image in Fig 7(e)] and higher rise with time after the first minute or so [areas of higher slope within the image in Fig. 7(b)]. The characteristics of the fitting can be tailored to extract the slope, and the model estimates in Fig. 6 can provide the calibration to the resulting extraction fraction in the tumor tissue from the capillary leakage. There is high variability in these values between tumors and within the tumor. For example, the range seen within the tumor for Fig. 7 was 0.02 up to 0.12, so there is significant value in being able to visualize the entire tumor surface as an image, as in Fig. 7. It is possible that the contrast of the slope map itself has a diagnostic surgical guidance value. Further studies are ongoing to determine the value of these images in the diagnostic estimation of the extraction fraction.

Although not presented here, results from imaging mice tumors through uncut skin appeared much different than those reported here. The ICG kinetic curves in which normal tissue was overlying the tumor tissue were nearly identical to normal skin, producing a ratio curve with zero slope. Thus, early in the study, we concluded that imaging directly on the exposed tumor surface was necessary for visualization of kinetics of the tumor itself. It is possible that short wave infrared (SWIR) imaging^30^^–^^32^ may provide deeper tissue penetrance or use of tissue clearing methods^33^^–^^35^ could provide deeper penetrance with NIR imaging, and these could be used in combination with the proposed model-based interpretation of vascular extraction mapping proposed here.

## 5 Conclusions

The summary of this exploratory work showed that the time kinetics of tissue ICG fluorescence intensity may have value in characterizing the tumor microvasculature. In particular, after bolus injection, 1 to 2 min of the observed tissue intensity kinetics appear to be most useful in predicting the capillary leakage, differentiating two pancreatic cancer tumor models with well-characterized differences in their neovascular patterns. The first minute was dominated by whole body recirculation effects from the bolus injection, whereas the latter timepoints appeared to wash out any strong differences between the two tumor lines. This study focused on the measurements and a model-based interpretation of them and did not complete any validation of the leakage rates via an independent measurement. One critically important factor in being able to accurately interpret the kinetics appears to be the normalization to some relevant normal tissue as an internal control measure of the body physiology and bolus injection response. A benefit of this process and the observations here is that the data processing can be extended to whole field imaging via image processing of the entire field of view, as shown in Fig. 7. Further study of these spatial maps and their accuracy and variability is certainly warranted.

The relevance of this paper to clinical use is still fairly speculative; however, the results do indicate that capillary leakage rate can be quantified with the process outlined here and that this leakage rate is somewhat indicative of the tumor type. The accuracy of any individual measurement has not been validated here, but further analysis to confirm the accuracy of this method should be carried out next. It seems likely that the capillary leakage rate would have value in predicting drug delivery and inflammation magnitude or potential. *In vivo* imaging of these features might have significant value from the spatial extent as well, given the well-known high heterogeneity of pancreatic cancers. The relevance to pancreatic cancer is particularly important given the bleak clinical outcomes and the need for further improvements in both invasive and systematic treatments. In addition, the techniques developed here may also have bearing upon imaging capillary leakage with other diagnostic modalities, such as computed tomography (CT) or magnetic resonance imaging (MRI). Further pre-clinical studies examining the value of capillary leakage rate imaging to treatment efficacy would likely be a good next step in this work.

## Data Availability

Data and code developed in this study are available upon reasonable request to the corresponding author.
